# Seizure Phenotype and Underlying Cellular Defects in *Drosophila* Knock-In Models of DS (R1648C) and GEFS+ (R1648H) *SCN1A* Epilepsy

**DOI:** 10.1523/ENEURO.0002-21.2021

**Published:** 2021-09-20

**Authors:** Alexa Joanna Roemmich, Thy Vu, Tamas Lukacsovich, Charlesice Hawkins, Soleil S. Schutte, Diane K. O’Dowd

**Affiliations:** 1 Department of Developmental and Cell Biology, University of California, Irvine, Irvine, California 92697; 2 Faculties of Medicine and Natural Sciences, Brain Research Institute, University of Zürich, CH-8057 Zürich, Switzerland; 3 Office of Intramural Training and Education, National Institutes of Health, Bethesda, Maryland 20892; 4 Department of Anesthesiology, University of Florida, Gainesville, Florida 32610

**Keywords:** *Drosophila*, electrophysiology, epilepsy, *SCN1A*, sodium channel

## Abstract

Mutations in the voltage-gated sodium channel gene *SCN1A* are associated with human epilepsy disorders, but how most of these mutations alter channel properties and result in seizures is unknown. This study focuses on two different mutations occurring at one position within *SCN1A*. R1648C (R-C) is associated with the severe disorder Dravet syndrome, and R1648H (R-H), with the milder disorder GEFS+. To explore how these different mutations contribute to distinct seizure disorders, *Drosophila* lines with the R-C or R-H mutation, or R1648R (R-R) control substitution in the fly sodium channel gene *para* were generated by CRISPR-Cas9 gene editing. The R-C and R-H mutations are homozygous lethal. Animals heterozygous for R-C or R-H mutations displayed reduced life spans and spontaneous and temperature-induced seizures not observed in R-R controls. Electrophysiological recordings from adult GABAergic neurons in R-C and R-H mutants revealed the appearance of sustained neuronal depolarizations and altered firing frequency that were exacerbated at elevated temperature. The only significant change observed in underlying sodium currents in both R-C and R-H mutants was a hyperpolarized deactivation threshold at room and elevated temperature compared with R-R controls. Since this change is constitutive, it is likely to interact with heat-induced changes in other cellular properties to result in the heat-induced increase in sustained depolarizations and seizure activity. Further, the similarity of the behavioral and cellular phenotypes in the R-C and R-H fly lines, suggests that disease symptoms of different severity associated with these mutations in humans could be due in large part to differences in genetic background.

## Significance Statement

Knock-in *Drosophila* lines were generated by CRISPR-Cas9 gene editing to explore how the R1648C (R-C) and R1648H (R-H) mutations in the *SCN1A* sodium channel gene contribute to distinct epilepsy disorders, Dravet syndrome, and GEFS+, respectively. *Drosophila* heterozygous for the R-C or R-H mutation displayed spontaneous seizures exacerbated at high temperature. GABAergic neurons in both mutants exhibited sustained depolarizations, interrupting neuronal firing, that increased in incidence at elevated temperature. A hyperpolarized deactivation threshold in R-C and R-H sodium currents was constitutive, implicating interaction with other heat-sensitive processes to alter firing properties. The similarity of the behavioral and cellular phenotypes in the R-C and R-H fly lines suggests that disorders of different severity in humans could be due in large part to differences in genetic background.

## Introduction

Mutations in voltage-gated sodium channels are implicated in a range of human seizure disorders (Escayg and Goldin, 2010). In the sodium channel gene *SCN1A,* there are >1250 dominantly inherited mutations identified in patients with epilepsy. Though the loci of these mutations are known, how individual mutations lead to patient symptoms is largely unknown.

The Na_V_1.1 sodium channel, encoded for by *SCN1A*, is composed of four homologous domains (I–IV), each with six transmembrane-spanning segments (termed S1–S6; Catterall, 2017). The fourth segment of each domain (S4) has conserved positively charged amino acids at every third position, notably arginines, which are important in activating the channel in response to depolarizing membrane potential (Shen et al., 2017; Pan et al., 2018). The S4 segment in domain IV is unique in that its movement is slower, and the movement uncovers a binding site for the inactivation loop in the pore (Chen et al., 1996; Chanda and Bezanilla, 2002; Goldschen-Ohm et al., 2013). In this study, we focused on two mutations in the S4 segment of domain IV, R1648H (R-H) and R1648C (R-C). Although the mutations occur at the same amino acid location within the channel, they lead to different forms of epilepsy. R1648H causes generalized epilepsy with febrile seizures plus (GEFS+) in patients (Baulac et al., 1999), while R1648C leads to Dravet syndrome (DS; Ohmori et al., 2002; Striano et al., 2008). GEFS+ is usually a moderate form of epilepsy, characterized by febrile seizures that persist beyond the age of 6 years. DS is generally more severe and is characterized by delayed development and impaired motor functions in addition to seizures. Both types of epilepsy feature febrile as well as spontaneous seizures. In addition, patients with these types of epilepsy are frequently resistant to anticonvulsant drugs (Ogiwara et al., 2007; Escayg and Goldin, 2010). As the connection between individual mutations and patient symptoms remains largely unknown, we aim to model patient-specific epilepsies in the laboratory.

Studies of isolated sodium currents in oocytes, HEK cells, and computational models found that the R1648H mutation resulted in a decreased use dependence and faster recovery from inactivation. In addition, both R1648H and R1648C mutant sequences resulted in large persistent sodium currents during sustained depolarization not seen in the wild-type channels (Spampanato et al., 2001; Lossin et al., 2002; Rhodes et al., 2004; Vanoye et al., 2006; Kahlig et al., 2010). Later studies using a knock-in mouse model of R1648H demonstrated that this mutation resulted in animals that displayed reduced threshold to heat-induced seizures. Analysis of isolated neurons indicated decreased repetitive firing selectively in bipolar inhibitory neurons, which has been attributed to reduced sodium currents with slower recovery from inactivation and increased use dependence inactivation (Tang et al., 2009; Martin et al., 2010). Surprisingly, there were no changes in persistent current levels, which had been a key result from heterologous expression system studies. These findings demonstrate that a single mutation can result in distinct underlying mechanisms of cellular dysfunction in heterologous expression systems and excitable cells. However, since there is no R1648C mouse model to date, it has not been possible to explore similarities and differences in changes associated with R-H versus R-C in mouse neurons. In addition, it is unknown how temperature affects channel function for either mutation.

To evaluate the effect of the same mutation at a single locus in excitable cells, and to explore the effects of temperature on both cellular and behavioral phenotypes, we used the *Drosophila* model. One advantage of using *Drosophila* is that they have only a single sodium channel gene compared with mammals, which have multiple genes encoding voltage-gated sodium channels including three that are highly expressed in the adult brain (*SCN1A*, *SCN2A*, and *SCN8A*; Escayg and Goldin, 2010). The *Drosophila* voltage-gated sodium channel gene (*para*) encodes a protein (Para) similar in structure and function to Na_V_1.1 (Loughney et al., 1989; O’Dowd et al., 1989). Previous studies showed that knock-in animals homozygous for the *SCN1A* mutation K1270T causing GEFS+ or S1231R causing DS displayed heat-induced seizure phenotypes with distinct properties in the two mutant lines that were not seen in the controls (Sun et al., 2012; Schutte et al., 2014, 2016). It was also noted that the mutations caused distinct alterations to sodium channel activity in homozygous neurons. In the present study, CRISPR-Cas9 gene editing was used to insert R1648C, R1648H, or R1648R (R-R) into the *Drosophila* sodium channel gene. Behavioral and electrophysiological studies were performed on R-C and R-H heterozygous mutants and R-R controls.

## Materials and Methods

### *Drosophila* generation

A plasmid expressing the DsRed marker fused to an eye-specific promoter and flanked by worm sequences and two arms (2 and 1.4 kb) homologous to *para*, as well as plasmids expressing guide RNA (gRNA), were injected into fly embryos that expressed Cas9 under the germ cell promoter *vas*. Successfully transformed adults were selected based on DsRed expression in their eyes. The *para* gene is located on the X chromosome in *Drosophila*, so disruption of the *para* gene in DsRed flies resulted in lethality in hemizygous males and homozygous females. The line was maintained by balancing the mutant X chromosome with FM7.

In the second step, a plasmid that expresses gRNA targeted to the worm sequences flanking DsRed, along with a plasmid containing the *para* gene exon 30 containing a point mutation, were injected into *vas*-Cas9 embryos. The repair template carried at the 1648 aa codon position either the desired mutation R-C (CGA to TGT) and R-H (CGA to CAT) or a silent mutation R-R (CGA to CGT) that serves as our control. Injected fly embryos that matured may produce eggs with the restored but mutated *para* gene. Females were crossed with FM7/Y males.

For the R-C and R-R transformation, the presence of the mutated *para* gene rescued male lethality, so males with non-bar-shaped eyes (expressing mutated *para* on the manipulated X chromosome) were used as founders for line propagation and then were sequenced for genotype verification. For the R-H transformation, male lethality was not rescued. Female flies were screened for the loss of DsRed, then were used as founders and sequenced. Sequencing of ∼1.7 kb of DNA surrounding the targeted mutation site and gRNA cut sites confirmed the presence of the predicted base pair change in each line with no additional mutations, other than a few silent mutations where two different codons for the same amino acid were present in the population. Multiple, independent lines were obtained, backcrossed for five generations on a UAS-GFP; w+ background, and maintained. R-C and R-H mutations are homozygous lethal, so all lines are maintained as heterozygous stocks over the X chromosome balancer FM7.

### Seizure behavior test

To simulate febrile conditions that induce seizures in individuals with GEFS+ and DS, flies 1 d posteclosion were placed in groups of five in empty vials that were then immersed in a water bath, first at room temperature and then at high temperature for 2 min. An external bath temperature of 40°C was selected as it was the lowest temperature that induced clear seizure-like behavior in all mutant flies within the 2 min trial period. The maximum temperature inside the vial after 2 min was ∼33°C (see Fig. 2*B*). Video recordings of the assay were evaluated, and the number of flies seizing in each vial at 20 s intervals was determined. A seizure was defined as a fly exhibiting a whole-body twitch or jump, or falling onto its back or side, often accompanied by leg twitching or wing flapping. Researchers were blinded to genotype during analysis. Data from multiple experiments were used to determine the average probability of seizure for a fly of a certain genotype at any second during the assay.

### Life-span assay

Three groups of 10 newly eclosed female flies from both mutant lines, R1648C and R1648H, as well as the control line R1648R, were separated by genotype and placed in vials laid horizontally containing standard cornmeal-based food. The flies were housed under a 12 h light/dark cycle at 23°C. Fatalities were recorded every 1–3 d, and food vials were changed twice weekly. Survival curves were created using the Kaplan–Meier estimate and analyzed using the Mantel–Cox log-rank test.

### Electrophysiology

Whole-brain dissections were conducted on adult female mutant or control flies 2 d posteclosion. Briefly, the fly was anesthetized on ice for 1 min, then decapitated. The eyes were cut and mouth parts removed, and the rest of the head was placed into an external solution (described below) also containing a papain suspension. After 5 min, the brain was removed from the cuticle and connective tissue was removed. Neurons targeted for recording were located in the dorsal antennal lobe. Local neurons (LNs) were distinguished from projection neurons based on the soma size (∼10 vs 6 μm) and action potential amplitude (∼30–40 vs 2–5 mV).

Whole-cell sodium currents and depolarization-evoked action potentials were recorded with whole-cell pipettes of 9–11 MΩ. Sodium currents were recorded using a pipette solution containing the following (in mm): 102 CsOH, 102 d-gluconic acid, 17 NaCl, 0.085 CaCl_2_, 1.7 MgCl_2_, 8.5 HEPES, 0.94 EGT, and 25 ATP. The pH was adjusted to 7.2 with CsOH, and the osmolarity was adjusted to 232–230 mOsm. The external solution contained the following (in mm): 122 NaCl, 3.0 KCl, 1.8 CoCl_2_, 0.8 MgCl_2_, 5.0 glucose, and 10 HEPES. It also contained 2.5 mm tetraethylammonium (TEA), 1.0 mm 1-aminopyridine (4-AP), 20 μm (+)-tubocurarine, and 10 μm picrotoxin. The pH was adjusted to 7.2 with NaOH, and the osmolarity was adjusted to 250–255 mOsm/L. Evoked action potentials were recorded using the same internal solution, replacing the CsOH with 102 mm potassium gluconate, and the d-gluconic acid was removed. The external solution was the same except that the CoCl_2_ was replaced by CaCl_2_ and the TEA and 4-AP were removed. Spontaneous action potentials were recorded using the same internal and external solutions as for evoked action potentials, without the presence of blockers. The chamber was continuously perfused at 0.75–1.0 ml/min, and the temperature in the chamber was controlled and monitored using a CL-100 Bipolar Temperature Controller (Harvard Apparatus). Data were acquired with an Axopatch 200B amplifier (Molecular Devices), a Digidata 1322A digital-to-analog converter (Molecular Devices), a Dell computer (Dimension 8200), and pClamp9 software (Molecular Devices).

For evoked electrophysiological recordings, baseline activity was recorded at 22°C, activity at elevated temperature was recorded at 30°C, and activity postcooling was recorded after the chamber had returned to 22°C for 5 min. Spontaneous burst characteristics were analyzed for each trace at sample intervals 90–110, 190–210, and 290–310 s for baseline 22°C data, and 690–710, 790–810, and 890–910 s for postcooling 22°C data. For elevated temperature, 60 s of data during heating was analyzed, starting at 26°C for each cell and reaching an average of 29.5°C at the end of the 60 s.

### Statistics

Statistical significance was determined using ANOVA with Tukey’s *post hoc* test for multiple experimental groups. Paired *t* tests were used to determine significance between room temperature and high-temperature data within a single genotype. Data are shown as the mean ± SE.

## Results

### CRISPR-Cas9 generation of *Drosophila* carrying epilepsy-causing mutations

**Figure 1.**
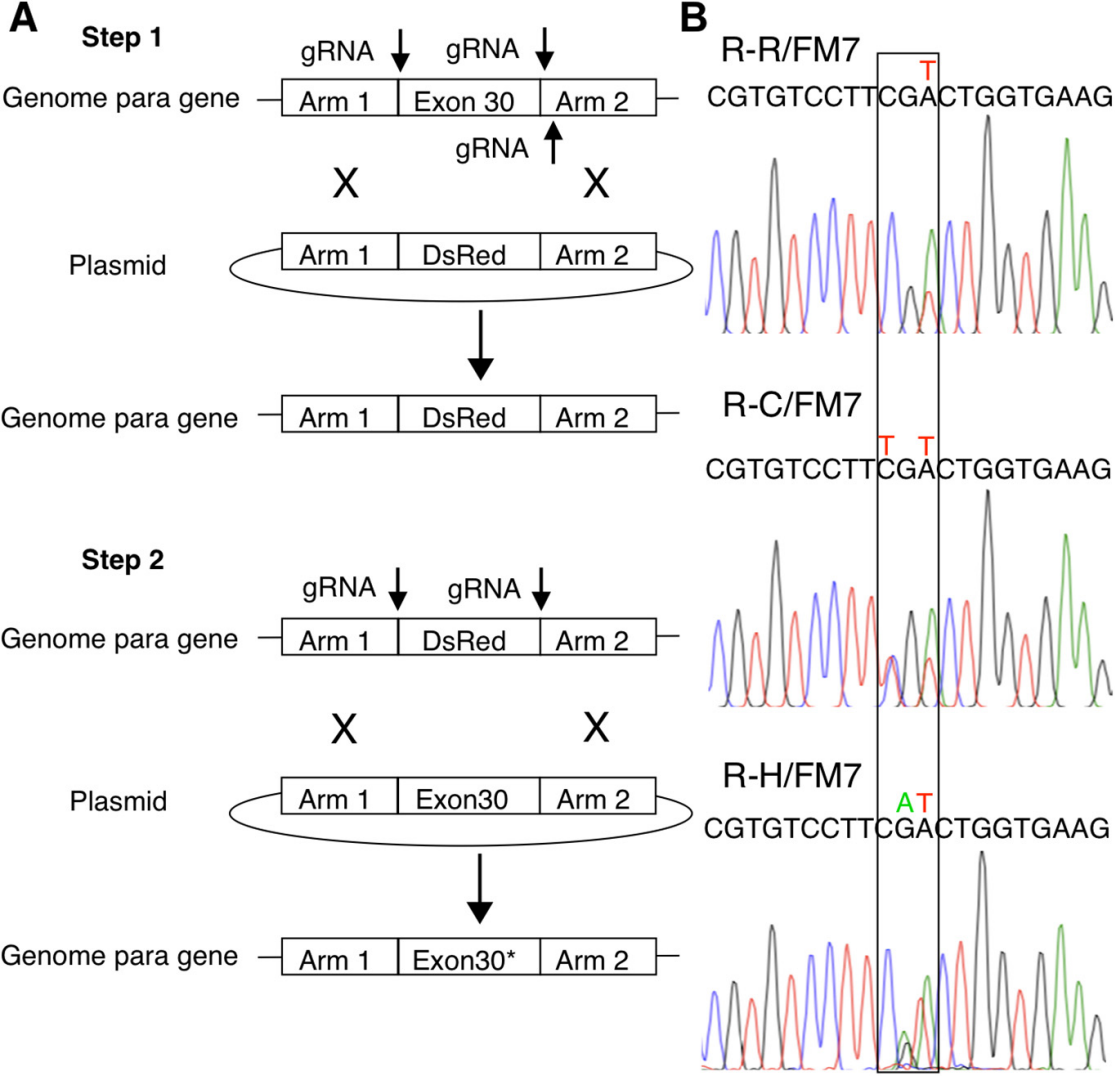
CRISPR strategy and validation. ***A***, Scheme of the two-step CRIPSR-Cas9-mediated genome editing for targeting the mutations to the *Drosophila para* gene. ***B***, Mutant *Drosophila* lines were confirmed by Sanger sequencing. The wild-type codon is CGA. Step 2 repair plasmids carried the silent mutation CGT for R-R controls, TGT for R-C mutants, and CAT for R-H mutants.

To create flies expressing patient-specific epilepsy-causing mutations, a two-step CRISPR-Cas9-mediated genome-editing strategy was used. In the first step, exon 30 of the *para* sodium channel gene, where the target missense mutations are located, was replaced by a DsRed marker flanked by arms including *Caenorhabditis elegans* sequences (Fig. 1*A*). In the second step, using gRNA targeted to the worm sequences, the DsRed marker and worm sequences were replaced by exon 30 of the *para* gene containing either the desired mutation R-C (CGA to TGT) and R-H (CGA to CAT), or a silent mutation R-R (CGA to CGT) that serves as the control (Fig. 1*A*). Sequencing of ∼1.7 kb of DNA surrounding the targeted mutation site and gRNA cut sites (Fig. 1*B*) confirmed the presence of the predicted base pair change in each line with no additional mutations. Multiple, independent lines were obtained, each resulting from a unique CRISPR insertion of R-C, R-H, or R-R. Both R-C and R-H mutations are homozygous lethal, so all lines were maintained as heterozygous stocks over the X-chromosome balancer FM7. Patients with these mutations are heterozygous, so all experiments were performed on heterozygous females.

### Mutants display seizure-like behavior and reduced life span

As individuals with DS and GEFS+ frequently have febrile-induced seizures in addition to spontaneous seizures, three fly lines heterozygous for the R-C mutation and four lines heterozygous for the R-H mutation were monitored for the presence of spontaneous and/or heat-induced seizure behavior. Groups of five flies were collected 1 d posteclosion and placed in a vial. A 2 min video was recorded immediately following immersion of the vial into a water bath, first at room temperature (22°C) and then at high temperature (40°C). Videos were later evaluated for seizure activity. A seizure was defined as the fly exhibiting a whole-body twitch or jump, or falling onto its back or side, often accompanied by leg twitching or wing flapping. Analysis was conducted blinded with respect to genotype.

**Figure 2.**
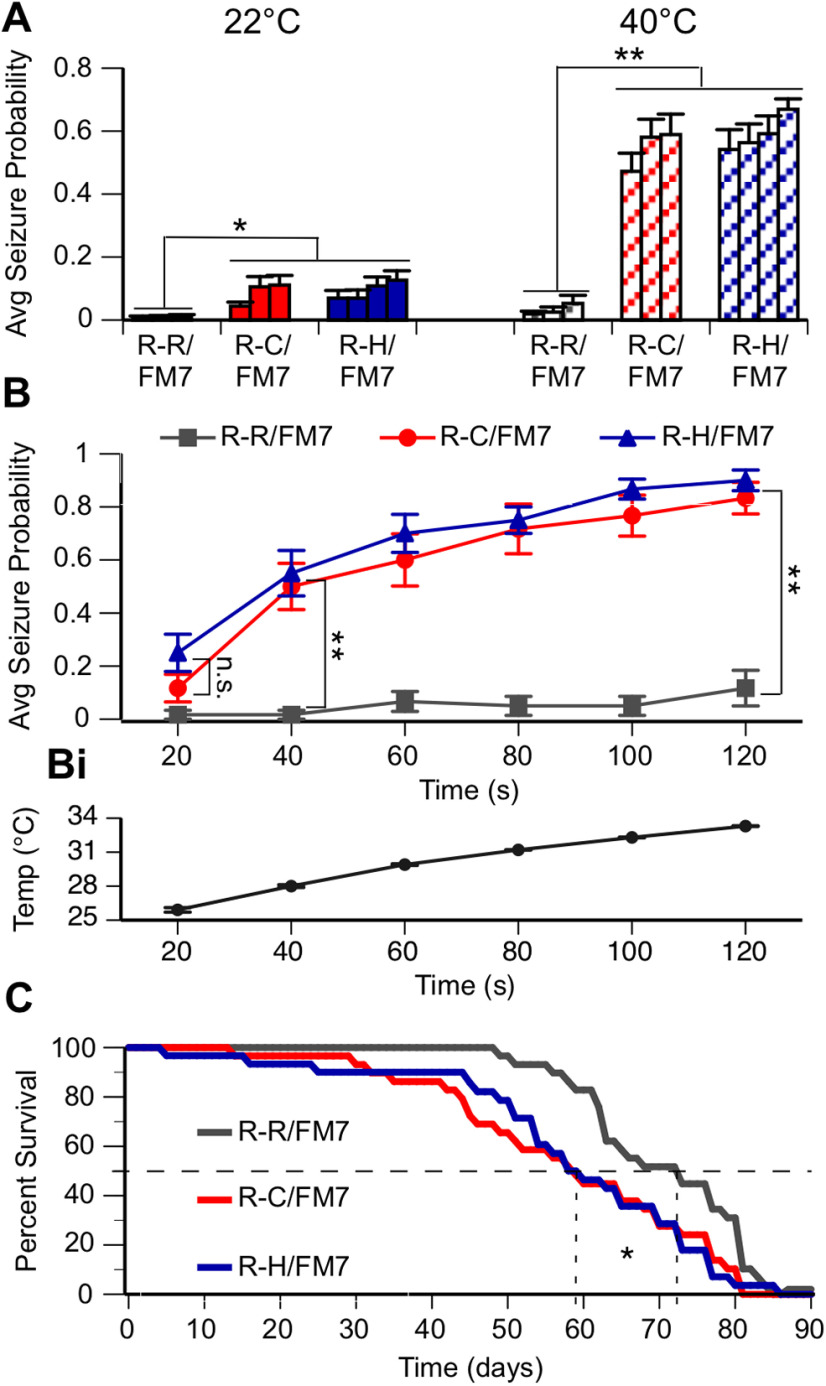
R-C and R-H mutants display seizure-like behavior and reduced life span. ***A***, Average seizure probability at any point in a 2 min trial for each independent line (3 R-R/FM7, 3 R-C/FM7, 4 R-H/FM7). ***B***, Average seizure probability for each genotype shown at 40°C over time. At both 22°C and 40°C, R-C and R-H mutants have a significantly greater average seizure probability compared with R-R controls. Comparisons between R-C and R-H flies or between lines of one genotype are not significant: *p < 0.05; **p < 0.01; two-way ANOVA, Tukey's *post hoc* test. *n* > 12 trials/group. ***Bi***, Average temperature inside vial over time of vial in 40°C bath. ***C***, Kaplan–Meier survival curve estimates of R-R, R-C, and R-H flies over time. Median survival (50%) line is shown. Median percentage of survival analyzed using the Mantel–Cox log-rank test. Life span is significantly longer in R-R flies compared with R-C and R-H flies. *p < 0.05. *n* = 30 flies/genotype.

Results are reported as the average probability of seizure for a fly of a certain genotype at any point in time during the assay. The four R-R control fly lines did not exhibit seizure activity in water baths at room temperature or high temperature (Fig. 2*A*). In contrast, both R-C and R-H heterozygous flies had a significantly increased seizure probability at room temperature compared with the R-R/FM7 control. At high temperature, seizure probability increased significantly in both R-C and R-H when compared with their behavior at room temperature, and with control R-R flies at high temperature (Fig. 2*A*; *p* < 0.05, ANOVA, Tukey’s *post hoc* test). There was no difference in seizure probability between lines with the same mutation. These data indicate that the seizure activity is associated with the introduced mutations, either R-C or R-H, and is not influenced significantly by the specific insertion events during the CRISPR process that resulted in the generation of each line.

To evaluate the time course of heat-induced seizure activity associated with the different mutations, multiple flies in single R-H, R-C, and R-R lines were examined at 20 s intervals over a 2 min period following immersion of the vial in water bath of 40°C. The actual temperature inside the vial was continuously monitored during heating, reaching a temperature of 33°C at 2 min (Fig. 2*Bi*). The onset of heat-induced seizures was similar in the R-C and R-H lines, with maximal seizure probability occurring between 100 and 120 s (Fig. 2*B*). The similarity in the spontaneous and heat-induced seizure phenotypes in the R-C and R-H lines was unexpected since the R-C mutation is associated with the more severe seizure disorder Dravet syndrome, while R-H is associated with GEFS+.

Our initial observations suggested that the mutant lines were more difficult to maintain over time than the control lines. Since seizure activity in humans can be associated with premature death and homozygous knock-in mice with the R-H mutation have reduced life span (Tang et al., 2009; Martin et al., 2010), this property was evaluated in the R-C, R-H, and R-R lines maintained at 22°C. Since individual lines from the same genotype displayed similar seizure phenotypes, one line from each genotype was chosen and was used exclusively for the life-span assay and the remainder of the studies. Both the heterozygous R-H and R-C flies display a markedly decreased median survival time of ∼60 d when compared with R-R flies with a median survival of 73 d (Fig. 2*C*; *p* < 0.05, Mantel–Cox log-rank test). This similarity in severity between R-C and R-H was again unexpected because the R-H mutation is associated with the less severe GEFS+ seizure disorder.

### Constitutive and heat-induced alterations in firing properties in R1648C and R1648H inhibitory neurons

Although behavioral studies facilitate an exploration of organismal dysfunction in the presence of R1648C and R1648H sodium channel mutations, studying neuronal firing and sodium current activity directly in the neurons of mutated flies is necessary to establish the underlying cellular mechanisms of dysfunction. In both mouse and fly knock-in models of *SCN1A* epilepsy, alterations in sodium currents and excitability have been primarily observed in inhibitory neurons (Ogiwara et al., 2007; Schutte et al., 2016). Therefore, we focused on evaluating the effects of R-C and R-H mutations in inhibitory neurons. Though the site of seizure generation in flies is unknown, LNs in the fly antennal lobes are a readily accessible population of GABAergic inhibitory neurons in the important and well characterized olfactory circuit within the *Drosophila* brain (Seki et al., 2010). To observe neuronal firing and sodium channel activity in these neurons, the whole brain was dissected out of adult flies (Roemmich et al., 2018), dorsal lateral local neurons were identified by their location and morphology, and whole-cell electrophysiological recordings were performed.

Evoked firing was evaluated by subjecting each cell to a series of depolarizing current steps, first at 22°C, following an increase in bathing solution temperature to 30°C, and finally after return of the bath solution to 22°C. A temperature of 30°C was chosen for high-temperature electrophysiology recordings as a temperature at which mutant flies exhibited seizure-like behavior during the water bath assay (Fig. 2*Bi*), and as the highest recording temperature at which quality cell recordings could be maintained. We measured the threshold, amplitude, half-width, and maximum rise slope for the first evoked action potential in each cell, the spike frequency adaption ratio (at half-maximal firing, first interspike interval over final interspike interval), and peak instantaneous firing frequency at a baseline of 22°C, during heating to 30°C, and after cooling back to 22°C for each genotype (Table 1). There were no significant differences between genotypes for any of these characteristics.

**Table 1:** Properties of evoked firing in R-R, R-C, and R-H LNs

	R-R/FM7	R-C/FM7	R-H/FM7
Input resistance, GΩ	0.64 ± 0.02	0.59 ± 0.10	0.56 ± 0.05
Cell capacitance, pF	20.32 ± 2.13	17.72 ± 2.01	21.13 ± 4.31
Action potential threshold, mV	22˚C: −52.45 ± 1.99	22˚C: −50.05 ± 3.81	22˚C: −50.68 ± 3.68
	30˚C: −42.71 ± 3.63	30˚C: −44.50 ± 5.26	30˚C: −46.40 ± 2.30
	22˚C: −50.25 ± 1.94	22˚C: −51.45 ± 4.88	22˚C: −50.13 ± 2.01
Action potential amplitude, mV	22˚C: 38.59 ± 2.70	22˚C: 33.65 ± 3.02	22˚C: 42.99 ± 2.91
	30˚C: 27.41 ± 3.58	30˚C: 23.32 ± 3.32	30˚C: 27.08 ± 3.87
	22˚C: 33.78 ± 3.40	22˚C: 35.67 ± 3.69	22˚C: 38.76 ± 4.19
Half-width, ms	22˚C: 3.0 ± 0.1	22˚C: 3.2 ± 0.2	22˚C: 2.8 ± 0.1
	30˚C: 2.2 ± 0.1	30˚C: 2.7 ± 0.3	30˚C: 2.6 ± 0.2
	22˚C: 2.6 ± 0.1	22˚C: 3.3 ± 0.3	22˚C: 3.1 ± 0.2
Max slope	22˚C: 24.78 ± 2.82	22˚C: 22.45 ± 4.17	22˚C: 29.32 ± 4.07
	30˚C: 27.97 ± 4.67	30˚C: 23.96 ± 4.88	30˚C: 26.27 ± 3.75
	22˚C: 22.18 ± 3.39	22˚C: 23.72 ± 5.91	22˚C: 25.31 ± 3.66
Adaptation ratio (ISI_first_/ISI_final_ at half-maximal firing)	22˚C: 0.49 ± 0.07	22˚C: 0.44 ± 0.09	22˚C: 0.56 ± 0.09
	30˚C: 0.27 ± 0.05	30˚C: 0.33 ± 0.08	30˚C: 0.33 ± 0.08
	22˚C: 0.45 ± 0.06	22˚C: 0.52 ± 0.09	22˚C: 0.64 ± 0.06
Peak instantaneous frequency, Hz	22˚C: 104.7 ± 12.4	22˚C: 111.5 ± 8.3	22˚C: 97.6 ± 9.2
	30˚C: 205.3 ± 22.8	30˚C: 237.8 ± 42.4	30˚C: 206.8 ± 35.6
	22˚C: 81.7 ± 10.5	22˚C: 90.7 ± 10.2	22˚C: 83.6 ± 10.2

Values are the mean ± SE at 22°C (during the 5 min preheating), at elevated temperature 30°C, and again at 22°C (during the 5 min postcooling). ISI, Interstimulus interval.

**Figure 3.**
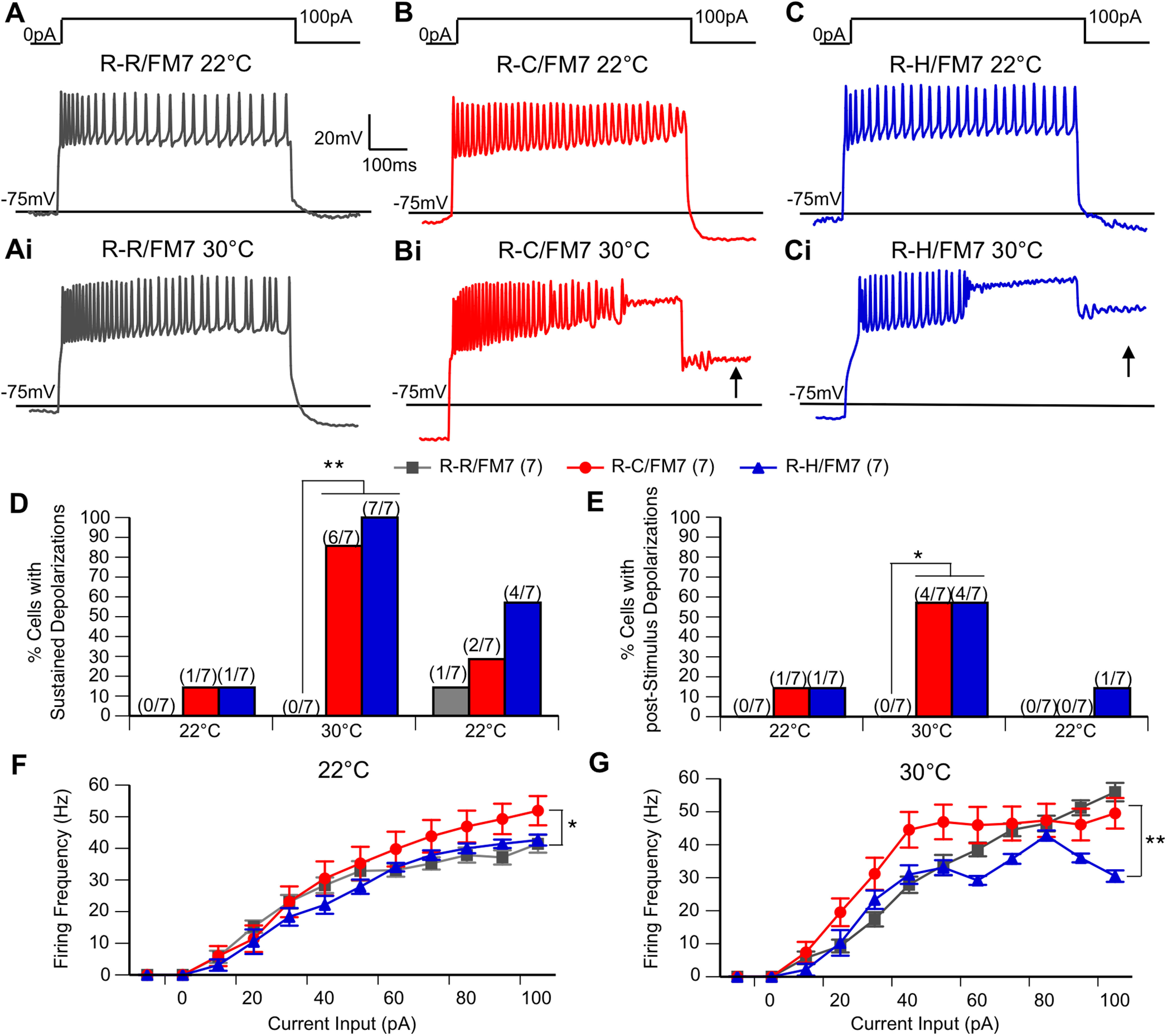
R-C and R-H mutants display sustained depolarizations during evoked firing, even after stimulus has ended. ***A–Ci***, Representative trains of action potentials evoked by illustrated stimulus protocol at 22°C (***A,B,C***) and 30°C (***Ai,Bi,Ci***). Arrow indicates the appearance of poststimulus depolarization. ***D***, ***E***, Percentage of cells in each genotype with sustained depolarizations during stimulus (***D***) and poststimulus depolarizations (***E***) at 22°C, at 30°C, and after cooling back to 22°C. R-C and R-H mutants have increased the incidence of SDs and poststimulus SDs at 30°C. *p < 0.05, **p < 0.01, χ^2^ test. Any differences before or after heating are not significant. ***F***, ***G***, Average spike frequency shown as a function of current input at ambient temperature (***F***) and elevated temperature (***G***). At 22°C, R-Cs have generally increased firing frequency. At 30°C, R-Hs have generally decreased firing frequency compared with R-Cs. *n* = 7 groups/genotype. *p < 0.05; **p < 0.01, two-way ANOVA, with Tukey’s *post hoc* test.

Before heating, the majority of neurons in all three genotypes fired action potentials throughout the 600 ms current step (Fig. 3*A*,3*B*,3*C*). While a similar firing pattern persisted in the control LNs (Fig. 3*Ai*) at 30°C, heating resulted in sustained depolarizations and cessation of action potential firing in R-C and R-H neurons. In some cases, depolarizations persisted after the current step (poststimulus depolarization; Fig. 3*Bi*,*Ci*). Quantitative analysis of these events demonstrates that at 22°C preheating, sustained depolarizations and poststimulus depolarizations occurred in a minority of mutant neurons but were never seen in control neurons (Fig. 3*D*,*E*). At 30°C, the incidence of sustained depolarizations increased dramatically in the mutant neurons such that nearly all of the R-C and R-H neurons exhibited these events (Fig. 3*D*). In contrast, there was no evidence of sustained depolarization or poststimulus depolarization in the control R-R neurons even at elevated temperature. Poststimulus sustained depolarization incidence increased as well, seen in the majority of mutant neurons at elevated temperature (Fig. 3*E*). The incidence of sustained depolarizations and poststimulus sustained depolarizations decreased postcooling, and there are no significant differences in these measures between genotypes at the return to 22°C. Importantly, the reversal of the temperature-induced changes in firing demonstrate that these alterations are associated with the mutation and are not simply a result of irreversible damage to the cell induced by heating.There were also differences in the firing frequency and response to temperature increase observed between control and mutant neurons. In control LNs, the firing frequency increased with higher current inputs at both 22°C and 30°C (Fig. 3*F*,*G*). At 22°C, R-C LNs displayed increased firing frequency compared with controls. However, for R-C and R-H LNs the firing frequency increased with higher current inputs only at 22°C. At 30°C, particularly for R-H LNs, the firing frequency began to decrease at the larger current steps corresponding with the appearance of sustained depolarizations in which the firing of action potentials stopped but the cell remained depolarized, either for the remainder of the stimulus, or even after the current injection was over (poststimulus sustained depolarizations; Fig. 3*F*,*G*; *p* < 0.05, two-way ANOVA).

**Figure 4.**
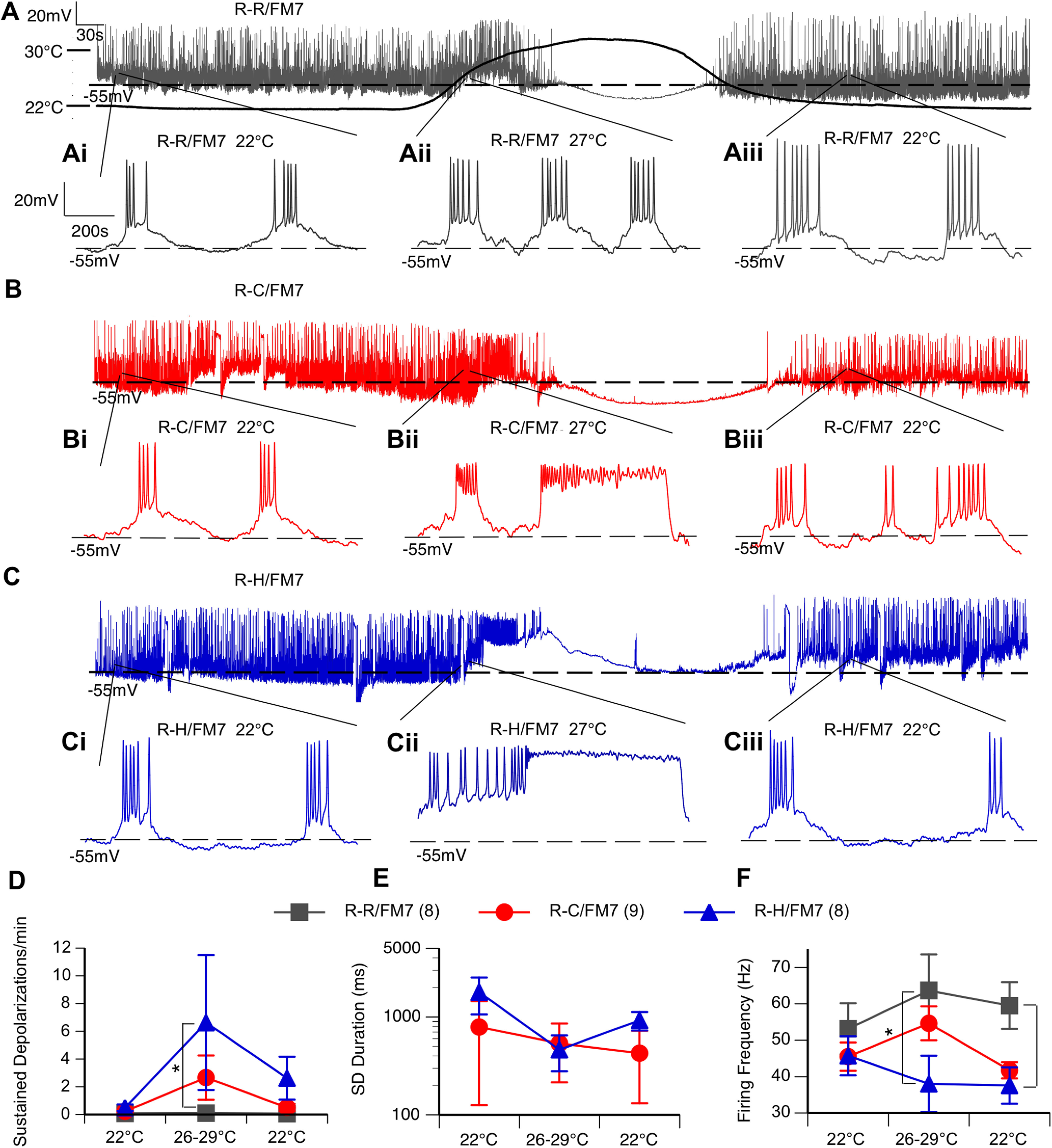
R-C and R-H mutants display spontaneous sustained depolarizations and reduced inhibitory firing frequency at elevated temperatures. ***A***, ***B***, ***C***, Representative continuous recording of neuron subjected to heating protocol. Chamber temperature over heating protocol shown on ***A***. ***Ai–Aiii***, ***Bi–Biii***, ***Ci–Ciii***, The 1.1 s interval within the labeled part of the temperature curve at 22°C preheating (***Ai***, ***Bi***, ***Ci***), 27°C mid-heating (***Aii***, ***Bii***, ***Cii***), and 22°C postcooling (***Aiii***, ***Biii***, ***Ciii***). ***D–F***, Average frequency and duration of sustained depolarizations (***D***, ***E***) and firing frequency of action potentials (***F***) during baseline (22°C), heating (26–29°C), and cooling (22°C). *n* = 8–9 cells/genotype. *p < 0.05, one-way ANOVA, with Tukey’s *post hoc* test.

Although evoked firing frequency in mutant LNs is generally comparable to that in controls, the presence of sustained depolarizations in the mutants could cause overall decreased excitability in interneurons. Therefore, we evaluated the spontaneous activity in LNs in the absence of any current injection. To explore the effect of temperature, spontaneous neuronal activity in each cell was recorded continuously for 14 min, as follows: 5 min at 22°C, 4 min during heating to 30°C, and 5 min during cooling back down to 22°C. The long traces in Figure 4*A*,4*B*,4*C* represent typical responses to the heating curve from each of the three genotypes. Demonstrated by the expanded timescale in Figure 4*Ai*, the traces consist of underlying oscillations in membrane potential with action potentials that fire at the peak of each oscillation. Both control and mutant neurons exhibited a regular burst firing pattern at 22°C (Fig. 4*Ai*,4*Bi*,4*Ci*) that ceased during heating at ∼30°C (Fig. 4*Aii*,*Bii*,*Cii*). Elevated temperature data were thus taken during the minute of heating between 26°C and 29°C (Fig. 4*D–F*). Burst firing similar to that seen before heating resumed following the return to 22°C, as illustrated in Figure 4*A*,*B*,*C* and *Aiii*,*Biii*,*Ciii*.

There were no differences in the average frequency of bursts, action potential threshold, action potential amplitude, or average number of action potentials within these bursts between genotypes (Table 2). There was also no significant effect of temperature on any of these properties.

**Table 2:** Properties of spontaneous burst firing in R-R, R-C, and R-H LNs

	R-R/FM7	R-C/FM7	R-H/FM7
Burst frequency, Hz	22˚C: 0.70 ± 0.24	22˚C: 0.90 ± 0.26	22˚C: 0.70 ± 0.24
	27˚C: 0.63 ± 0.26	27˚C: 0.57 ± 0.17	27˚C: 0.63 ± 0.26
	22˚C: 0.57 ± 0.20	22˚C: 0.37 ± 0.16	22˚C: 0.57 ± 0.20
Action potential threshold, mV	22˚C: −37.77 ± 1.48	22˚C: −37.48 ± 1.83	22˚C: −38.00 ± 2.45
	27˚C: −36.74 ± 1.36	27˚C: −37.06 ± 1.77	27˚C: −33.81 ± 3.10
	22˚C: −36.79 ± 1.66	22˚C: −35.95 ± 2.08	22˚C: −36.17 ± 2.40
Action potential amplitude, mV	22˚C: 35.34 ± 2.35	22˚C: 32.27 ± 3.83	22˚C: 28.18 ± 5.16
	27˚C: 28.29 ± 2.34	27˚C: 23.16 ± 3.19	27˚C: 23.33 ± 3.87
	22˚C: 34.27 ± 3.81	22˚C: 28.52 ± 2.57	22˚C: 31.33 ± 4.16
Action potentials per burst	22˚C: 5.27 ± 0.39	22˚C: 5.24 ± 0.56	22˚C: 4.79 ± 0.35
	27˚C: 5.46 ± 0.38	27˚C: 5.05 ± 0.56	27˚C: 4.34 ± 0.87
	22˚C: 6.17 ± 0.50	22˚C: 5.31 ± 0.39	22˚C: 5.23 ± 0.49
Resting membrane potential, mV	22˚C: −57.1 ± 2.2	22˚C: −54.2 ± 2.0	22˚C: −54.0 ± 2.6
	27˚C: −55.9 ± 1.8	27˚C: −52.1 ± 1.3	27˚C: −47.8 ± 3.2^*^
	30˚C: −57.4 ± 2.4	30˚C: −58.3 ± 4.0	30˚C: −47.9 ± 1.9^*^
	22˚C: −54.9 ± 3.2	22˚C: −49.5 ± 2.1	22˚C: −49.8 ± 1.8

Values are the mean ± SE at 22°C (during the 5 min preheating), “27°C” (during the minute of heating from 26°C to 29°C), and again at 22°C (during the 5 min postcooling).

* R-H mutants have depolarized RMP at elevated temperatures compared with R-R controls; *p* < 0.05, two-way ANOVA.

However, the occurrence of spontaneous sustained depolarizations (>100 ms in duration) was a characteristic of both mutant lines (Fig. 4*Aii*–*Cii*,*D*,*E*). While both R-C and R-H mutants occasionally exhibited sustained depolarizations at room temperature (Fig. 4*E*), the average frequency of sustained depolarizations was increased at elevated temperatures in both mutants, but the increase was only significantly different in the R-H mutants compared with controls (Fig. 4*D*; *p* < 0.05, two-way ANOVA). The duration of the sustained depolarizations was similar in the two mutants, and the duration did not further increase with temperature (Fig. 4*E*). These prolonged sustained depolarizations contributed to decreased firing frequency within bursts for R-H neurons, particularly at elevated temperature (Fig. 4*E*; *p* < 0.05, two-way ANOVA).

At elevated temperatures, resting membrane potential (RMP) first depolarized, then hyperpolarized above 30°C as cells ceased firing. While RMP did not fully recover over the length of the recording, R-H neurons displayed a significant depolarizing shift in resting membrane potential at elevated temperatures (Table 2; two-way ANOVA).

### Alterations in sodium currents in R1648C and R1648H inhibitory neurons

**Figure 5.**
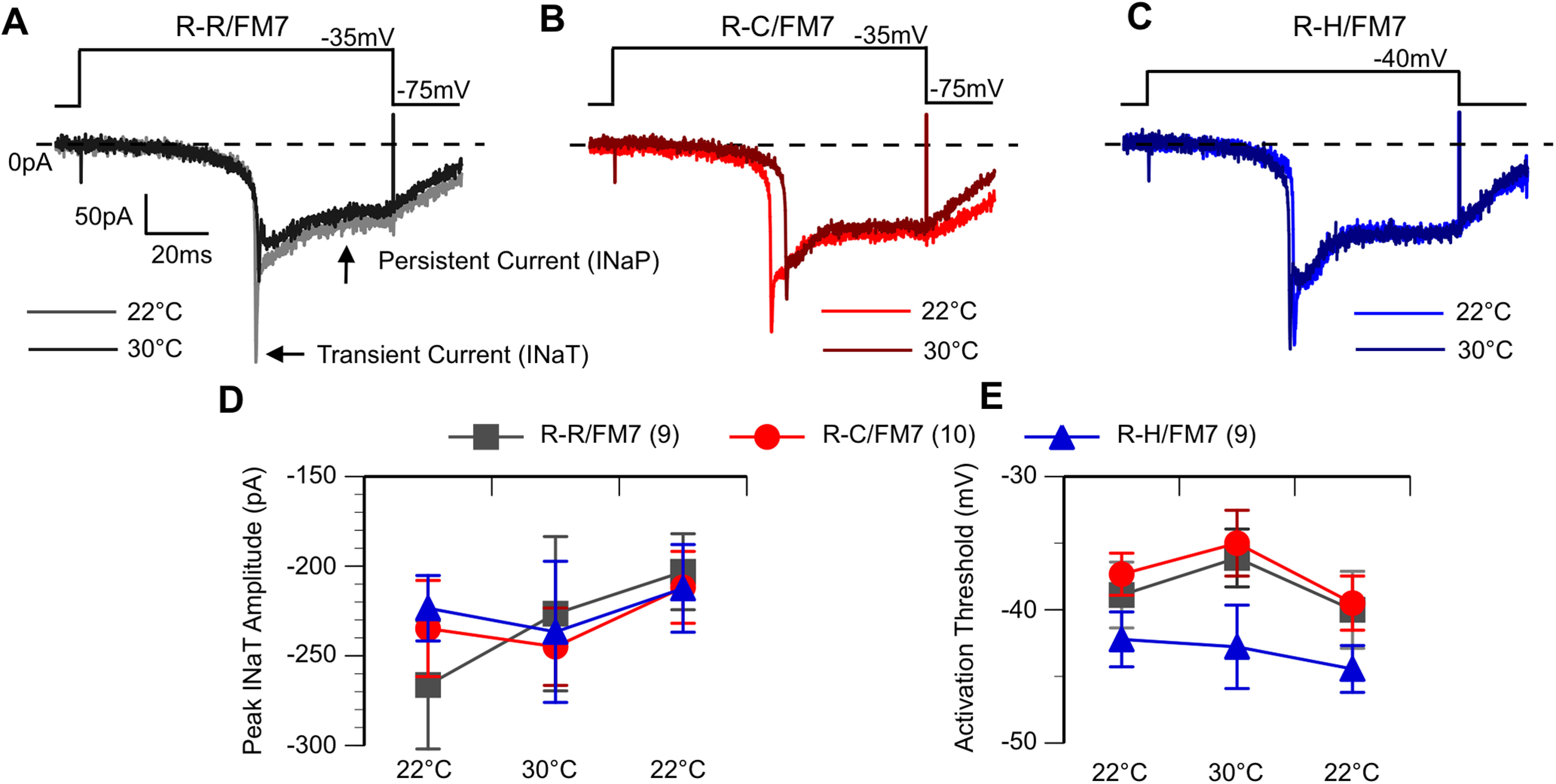
R-H neurons exhibit hyperpolarized sodium current activation threshold. ***A–C***, Voltage step-elicited currents at 22°C and 30°C. The first voltage step to elicit current shown. Cells exhibit both an initial transient and then a persistent current. ***D***, Average peak INaT amplitude. ***E***, Average activation threshold at 22°C, 30°C, and following cooling (second threshold at 22°C). R-H mutants have an average activation threshold hyperpolarized to R-C and R-R, but no effect by temperature. *p < 0.05, two-way ANOVA, with Tukey’s *post hoc* test.

To evaluate changes in the underlying sodium currents, voltage-clamp recordings were conducted on inhibitory neurons in R-R, R-C, and R-H flies at both ambient and elevated temperatures. Sodium currents were activated by holding the cell below firing threshold at −75 mV and applying a depolarizing voltage step in increasing 5 mV steps. The sodium current waveform is similar in all three cells lines with an initial transient current (INaT) and a smaller persistent current at both 22°C and 30°C (Fig. 5*A*). The significant delay between the stimulus onset and sodium current activation at all recording temperatures is because of the recording electrode being located on the LN cell body, which is electronically distant from the sodium channels located on the distal axonal segments in fly neurons (Ravenscroft et al., 2020). While there were no genotype- or temperature-induced changes in latency between stimulus and response onset, this recording configuration prohibits adequate space clamp, preventing accurate assessment of the time course of activation and inactivation. Additionally, the transient sodium current amplitude in many neurons showed a decrease during the heating regime (Fig. 5*A–C*) that did not recover when cooled (Fig. 5*D*). Therefore, we did not further evaluate the potential effects of the mutation on peak current amplitude. In contrast, the average activation threshold, defined as the least depolarized voltage step that activates the first current, was stable throughout the recording. This property was significantly more hyperpolarized in R-H compared with R-C and R-R, but there was no effect of temperature (Fig. 5*E*; *p* < 0.05, two-way ANOVA).

**Figure 6.**
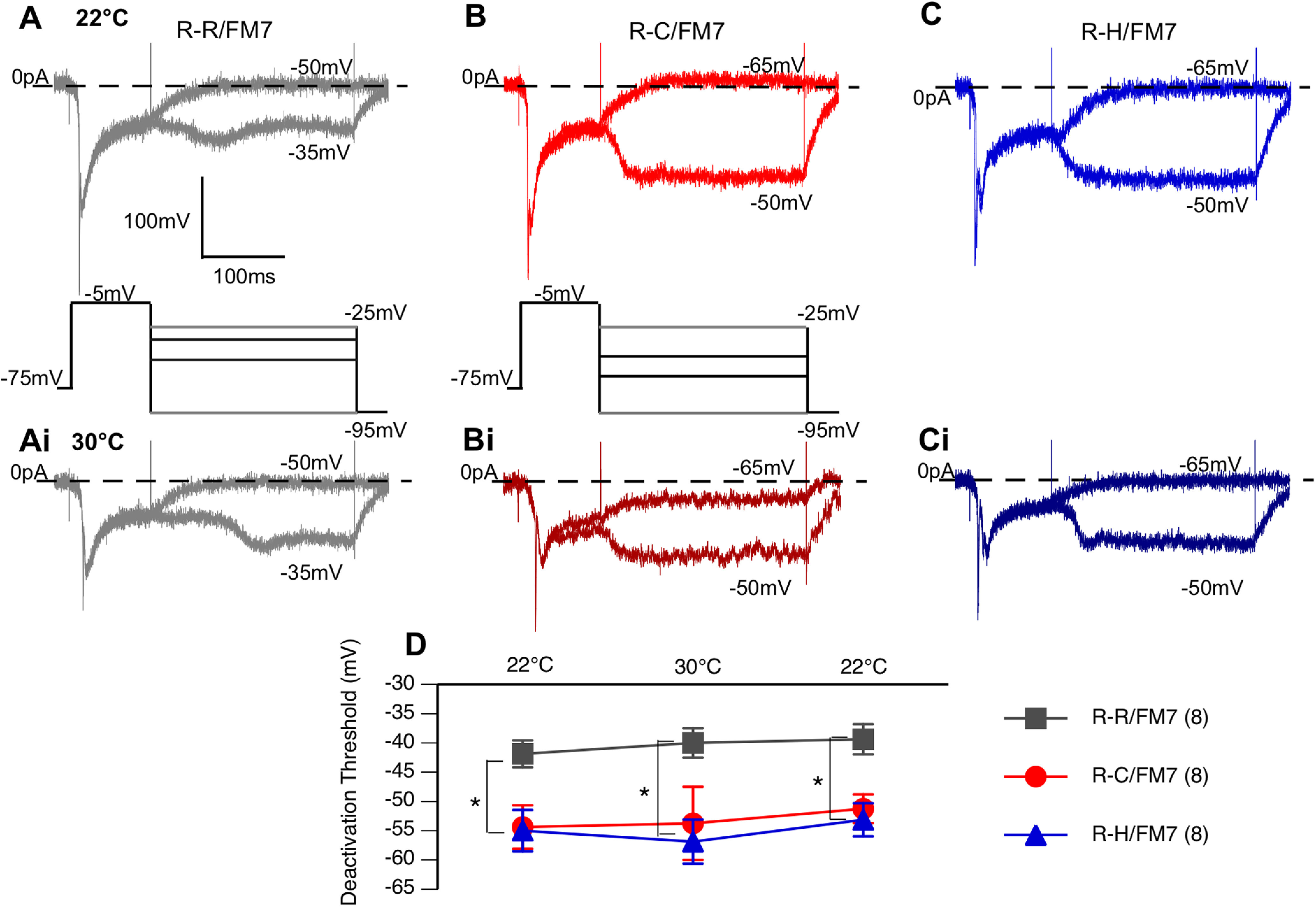
R-C and R-H neurons exhibit hyperpolarized sodium current deactivation threshold. ***A–Ci***, Voltage-step sodium channel currents for deactivation at room temperature (22°C; ***A***, ***B***, ***C***) and high temperature (30°C; ***Ai***, ***Bi***, ***Ci***), with the current steps in bold. Cells subjected to a multistep protocol, as shown. Selected sweeps shown to illustrate differences in deactivation properties. ***D***, Average deactivation threshold at 22°C, 30°C, and after cooling back to 22°C. R-C and R-H mutants had hyperpolarized deactivation threshold compared with R-Rs. *p < 0.05, two-way ANOVA, with Tukey’s *post hoc* test.

To examine the current deactivation properties, a multistep protocol was used. The cell was first depolarized to a fixed voltage of −5 mV to activate sodium currents and then returned to different resting potentials that varied in 10 mV steps between −95 and −25 mV (Fig. 6*A–Ci*). Deactivation threshold was defined as the least negative voltage step resulting in current returning to baseline at the end of the stimulus. Both the R-C and R-H neurons had a significant hyperpolarizing shift in deactivation threshold (−55 mV) compared with that of R-R control neurons (−40 mV; Fig. 6*D*; *p* < 0.05, two-way ANOVA). There was no effect of temperature on the deactivation. The deactivation data indicate that mutant sodium channels in inhibitory neurons are open over a wider voltage range, resulting in increased influx of sodium ions for the same stimulus in mutants compared with controls. However, unlike the abnormal firing properties, the sodium current alterations in deactivation were present at similar levels at both room temperature and high temperature.

## Discussion

For patients with genetic epilepsy, the connection between a specific mutation and behavioral symptoms is largely unknown. Our study focused on *SCN1A*-mediated genetic epilepsies that arise from mutations at the same amino acid position (1648). The R-C mutation is associated with the more severe disorder Dravet syndrome, while the R-H mutation is associated with the milder disorder GEFS+ (Baulac et al., 1999; Ohmori et al., 2002; Striano et al., 2008; Escayg and Goldin, 2010). This leads to the question of whether the differences in severity arise from distinct changes in channel function in the two mutants or whether similar channel changes result in different behavioral outcomes depending on genetic background. Using independently generated *Drosophila* lines carrying either the R-H or R-C mutation, we were able to compare the effects of the mutations on cellular physiology and organismal behavior in an identical genetic background. We observed that both R1648C and R1648H mutations resulted in largely similar spontaneous and temperature-sensitive seizure activity, decreased life span, and several patterns of cellular dysfunction. This suggests that it is likely that genetic background contributes to difference in severity in patients with these mutations.

While animal behavior was essentially indistinguishable between R-C and R-H lines, there were instances of significant electrophysiological alterations in one, but not both, mutant lines. R-C mutants displayed increased evoked firing frequency at ambient temperatures. R-H mutants displayed decreased evoked firing frequency at elevated temperatures, decreased spontaneous firing frequency at elevated temperatures, depolarized resting membrane potential at elevated temperatures, and a constitutively hyperpolarized sodium channel activation threshold. While R-H local neurons displayed more significant differences from controls than R-C local neurons, these cellular changes surprisingly did not result in a more severe behavioral profile. It seems that the changes shared by both mutant lines—a hyperpolarized sodium current deactivation threshold and increased incidence of sustained depolarizations seen most clearly with evoked firing—have the greatest effect on the behavioral phenotypes in R-C and R-H *SCN1A Drosophila*.

Additionally, many of the changes attributed to the mutation were observed in only a minority of mutant neurons, such as the appearance of sustained depolarizations in only one of seven LNs in each of the mutant lines at 22°C. This is most likely because of this being a low-frequency event in any one neuron at this temperature. Thus, there is a low probability that an event will be detected during the one or two trials during which the data are collected. If this were true, then increasing the number of trials in each cell at 22°C would increase the percentage of LNs in which at least one event was detected. At high temperature, the majority of neurons in both mutant lines exhibit these events, demonstrating that the alteration that leads to these existed in all the neurons but was only manifest at high temperature. It is possible that larger sample sizes could even out some of these smaller changes between R-C and R-H mutants. It is also possible that there are different subclasses of LNs with distinct distributions of sodium channels in the cell, such that some are more susceptible to the mutation resulting in sustained depolarizations at 22°C.

Given that the R-C DS mutation did not result in increased behavioral severity in *Drosophila*, and, given that several smaller changes in R-H sodium channel physiology did not result in increased seizures in *Drosophila*, there could be other background alterations that contribute to disease phenotype in humans. A role for genetic background in contributing to disease phenotype is not inconsistent with clinical data indicating that the severity of seizure disorders can vary even between individuals with the same *SCN1A* mutation. For example, in a single multigenerational French family, R1648H was identified in 13 individuals whose symptoms ranged from experiencing a few febrile seizures in early childhood to intractable epilepsy throughout adulthood associated with moderate or severe intellectual disability (Baulac et al., 1999). This suggests that interplay between the mutation and other factors are involved in determining disease severity. This could include genetic background or environmental/developmental differences. Recent studies in human induced pluripotent stem cell-derived neurons demonstrate that the GEFS+ causing the K1270T mutation has differential effects on sodium currents and neuronal firing properties in different genetic backgrounds (Xie et al., 2020). Transgenic *Drosophila* will be valuable in assessing the interaction between specific epilepsy causing missense mutations and genetic background in manifestation not only of cellular dysfunction but also of behavioral phenotypes.

Previous studies using mice have been important in understanding cellular mechanisms associated with several epilepsy-causing sodium channel mutations, including a knock-in model of the missense mutation R1648H (Tang et al., 2009; Martin et al., 2010). While the phylogenetic distance between humans and flies is greater than that between humans and mice, there are some distinct advantages to conducting parallel studies in *Drosophila*. The DNA sequences that encode voltage-gated sodium channels are highly conserved between flies and mammals, but *Drosophila* has only one voltage-gated sodium channel gene (*para*; Loughney et al., 1989; O’Dowd et al., 1989). In contrast, there are nine different voltage-gated sodium channel α-subunit isoforms in mice and other mammals, three of which are highly expressed in the nervous system (*SCN1A*, *SCN2A*, and *SCN8A*; Catterall, 2017). These isoforms can and do interact in models of patient epilepsies. For example, mice that were double heterozygous mutants for *SCN1A* and *SCN8A* had restored seizure susceptibility and life span compared with mice heterozygous for only *SCN1A* (Martin et al., 2007). In addition, creation and maintenance of multiple different mouse lines carrying distinct missense mutations in different genetic backgrounds is a time- and resource-intensive proposition. In the present study, the transgenic flies used were created using a two-step CRISPR/Cas9 gene-editing method that involved introducing a visible marker in the first step. This resulted in efficient detection of multiple founder flies with the desired mutations in an identical genetic background. In addition, the low cost of maintaining multiple lines, including control substitutions in addition to the mutants, was an attractive reason to use *Drosophila*. While there is only one sodium channel gene in flies, worm sequences not present elsewhere in the genome flanked the replacement sequence in the first step targeted for excision in the second step. Sequencing through the excision sites revealed identical sequences where additional mutations were most likely to be introduced, decreasing any concerns about off-target effects. Further confirmation of the specificity of mutants could be done by determining whether a genetic rescue, such as using CRISPR to replace the mutation in the R-C or R-H lines, restores wild-type function.

Analysis of two previous *Drosophila* knock-in models of *SCN1A* missense mutations, one causing Dravet syndrome (S1231R) and one causing GEFS+ (K1270T) exhibited alterations in sodium current and firing properties only in animals homozygous for the mutations (Schutte et al., 2016). Heterozygotes exhibited moderate seizure behavior at high temperatures but no obvious alterations in neuronal activity (Sun et al., 2012; Schutte et al., 2014). Therefore, the finding that fly lines homozygous for the R-C or R-H mutations were lethal, and that heterozygotes displayed significant seizure activity and reduced life span, was somewhat unexpected. The location of the 1648 mutation on the voltage-sensing region of *SCN1A*, which is particularly crucial for inactivation, may explain this increased level of severity compared with the previously examined DS (S1231R) and GEFS+ (K1270T) mutations. The R-C mutation substitutes a neutral amino acid for a positive gating charge. R-H conserves charge, but adds steric hindrance. Interestingly, in a study investigating the effects of an R-H mutation in the S4 segment of domain IV of a human skeletal sodium channel expressed in HEK cells, changing the pH to protonate the histidine did not change the functional alterations of the mutation (mixed inactivation defects; Alekov et al., 2000). This suggests that the steric effects of the histidine side chain may be the primary molecular culprit behind the phenotype associated with the R1648H mutation. Changing the size and/or charge of one of the gating charges on the S4 segment in domain IV likely affects its ability to exchange ion-binding partners with the environment. Given our observations that R1648H or R1648C mutations in inhibitory neurons result in sustained depolarizations and require repolarization to more negative voltages to deactivate the sodium current, both mutations likely impact the ability of the S4 segment in domain IV to move back into place, closing the channel (Ahern et al., 2016). The S4 segment in domain IV of *SCN1A* may therefore play a previously undescribed role in deactivation, as well as inactivation.

The R-C and R-H inhibitory neurons exhibited heat-induced alterations in neuronal firing properties including the appearance of sustained depolarizations, poststimulus sustained depolarizations, and decreased firing frequency during both evoked and spontaneous activity. The temperature-dependent aspect of these changes suggests that they are likely to contribute to the heat-induced increases in seizure activity. Analysis of the underlying sodium currents in mutant neurons revealed a hyperpolarized deactivation threshold in R-C and R-H neurons even at room temperature. This is consistent with the R-H and R-C sodium channels being more difficult to close once activated, which would result in increased ion flow because of longer channel openings and/or channel opening to a greater extent. Dravet syndrome has most often been attributed to loss-of-function mutations, as was the case in the previous S1231R Dravet syndrome knock-in model in *Drosophila*. The current result indicates that mutations resulting in increased sodium influx can also contribute to Dravet syndrome. However, unlike the abnormal firing properties, mutant sodium current alterations were present at both room temperature and high temperature. This suggests that the increased inward flow of sodium ions is not sufficient to trigger the sustained depolarizations and reduced firing frequency seen at high temperature by themselves but through an interaction with a different and as yet unidentified heat-induced change.

One heat-induced aspect of cellular physiology that could be involved are calcium currents. Previous studies have found that calcium currents exhibit increased conductance at elevated temperature in rat brain slices (Radzicki et al., 2013). Previous studies have shown that calcium currents also contribute to membrane depolarization in *Drosophila* neurons, including in the neurons located in the adult fly brain (Peng and Wu, 2007; Gu et al., 2009). In projection neurons in the adult antennal lobes, the calcium currents have both a transient and sustained component. The *alpha1T* gene encodes a Ca_v_3-type calcium channel mediating the transient current (Iniguez et al., 2013), and the *cac* gene encodes a Ca_v_2-type calcium channel mediating the sustained current (Gu et al., 2009). If similar calcium currents are present in antennal lobe LNs, it is possible that potassium currents are sufficient in most cases for repolarizing the action potential, even with the increased sodium conductance in the mutants because of hyperpolarized deactivation of the channels at room temperature, resulting only rarely in sustained depolarizations. However, at high temperature an increased magnitude of calcium currents, in conjunction with an increased magnitude of the sodium currents in the mutants, could more severely impede the ability of potassium currents to repolarize effectively, resulting in more sustained depolarizations. Future studies, taking advantage of both forward and reverse genetic approaches that are relatively easy to perform in *Drosophila*, will be important in identifying the interacting variables associated with the heat-induced change in firing properties.

In the mouse model, the primary effect of the R1648H mutation is on inhibitory neurons, where a decrease in inhibitory neuron activity can lead to unbalanced overexcitability in the brain and seizures (Martin et al., 2010). The present study focused exclusively on LNs, inhibitory neurons in the antennal lobe that process olfactory information in *Drosophila*. While the para sodium channel in *Drosophila* undergoes extensive alternative splicing (Loughney et al., 1989; Lin et al., 2009), the R1648 amino acid is located in an obligate exon, so mutations at this site will be present in all neurons (Lin et al., 2009; Ravenscroft et al., 2020). Therefore, it will be interesting in future studies to also evaluate the effect of this mutation on excitatory neurons in the same antennal lobe circuit.

Together, the results of the present study demonstrate that R1648C and R1648H *SCN1A* mutations result in improper deactivation in inhibitory neuron sodium channels in *Drosophila*. The incomplete closing and/or extended opening of the sodium channels could cause an inappropriate influx of sodium ions, which in turn causes impaired inhibitory neuron firing. Neuronal network activity is based on a balance between inhibitory and excitatory activity, and with the impaired firing of inhibitory neurons, the balance is tilted in favor of overexcitation, which can lead to seizures. CRISPR-generated *Drosophila* lines represent low-cost platforms that can be used to elucidate neuronal mechanisms of seizure generation and guide the development of new patient-specific therapies for epilepsy. Patient-specific studies using model organisms, including *Drosophila*, will be an important tool in our quest to improve our mechanistic understanding of genetic epilepsy and develop better patient-specific treatments.

## Synthesis

Reviewing Editor: Viji Santhakumar, University of California, Riverside Decisions are customarily a result of the Reviewing Editor and the peer reviewers coming together and discussing their recommendations until a consensus is reached. When revisions are invited, a fact-based synthesis statement explaining their decision and outlining what is needed to prepare a revision will be listed below. The following reviewer(s) agreed to reveal their identity: MacKenzie Howard, Hakeem Lawal.

The study uses CRISPER-Cas9 to develop Drosophila mutants of SCN1A relevant to Dravet and GEFS+ epilepsy syndromes and characterizes the model. The reviewers are in agreement that the experiments are well-designed and rigorously conducted and provides new information regarding the basic physiology and behaviors which are of interest to the field. While the lack of mechanistic examination was noted as a concern, the thorough characterization of the novel model presented here was found to have considerable merit. There were, however, some specific concerns noted by the reviewers which need to be adequately addressed.

1. A more extensive reporting of the intrinsic active and passive properties of neurons under control and hyperthermic conditions is needed to enable correlating changes in sodium channel deactivation with neuronal firing. The potential for compensatory changes to maintain cell intrinsic physiology should be considered and presentation of the full complement of intrinsic physiology data would help identify potential compensatory changes.

2. While genetic or pharmacological rescue experiments demonstrating restoration of the WT state in knock-in mice would be ideal to ensure specificity, the authors should, minimally, discuss the need for such an experiment as a potential weakness.

3. In Fig. 3, differences in the firing phenotype between R-C and R-H variants and the variability in penetrance should be discussed.

4. The long duration traces in figure 4 need to be better explained. It is unclear what the lines signify and basal frequency and action potential characteristics appear different between genotypes and need to be quantified.

5. The apparent change in latency to response onset observed in Fig. 5 needs to be quantified and discussed.

6. The high temperature used (40 deg) should be adequately justified.

7. Several minor issues with the figures, raised by the reviewers, need to be addressed to improve clarity.

Reviewer 1:

In this work the authors have engineered novel models of two human epilepsies, Dravet syndrome and GEFS+, which can both be caused by mutations of the same gene but have different outcomes. They have accomplished this using CRISPR-Cas9 to insert point mutations into the Drosophila para gene. They have measured seizures, early mortality, and changes in single neuron excitability and sodium currents using whole cell electrophysiology. These experiments are well-designed and rigorous. While these experiments don’t reveal mechanistic differences between these two epilepsy syndromes, this work is worthy of publication after revision and will be of interest to the readership of eNeuro.

Major comments:

The first point on which this manuscript could be improved is a deeper analysis of data on their most important findings. The report changes in action potential firing under control and hyperthermic conditions (Fig 3 and 4), and changes in sodium channel deactivation that could underlie this (Fig 6). However, a great deal more data should be presented from these experiments (which does not require performing more experiments). Action potential rise, decay, spike peak amplitude, and spike half width, action potential voltage threshold, peak instantaneous frequency, and spike frequency adaptation would all be of interest and could help link functional changes in spike behavior with changes in sodium channel deactivation. Basic intrinsic properties such as input resistance and resting membrane potential, as well as these spike properties, compared between normo- and hyper-thermic conditions could indicate how sodium channel changes alter baseline excitability or how neurons may be compensating for altered activity. Neurons appear to hyperpolarize during hyperthermia (Fig 3 and 4). Is this a consistent change?

In Figure 4, the long duration recording traces (panels A-C) presented are difficult to interpret. Is baseline spontaneous AP frequency changed? What are the properties of those action potentials? Do the bursts describe in Table 1 have the same duration and number of spikes across genotypes? Both mutant examples appear to exhibit periods of depolarization at 22 degrees. Is this true of most or all mutant neurons?

Finally, in Fig 5 the authors mention the clear change in delay to onset of sodium current in mutant neurons during hyperthermia. This seems like an important change that should be quantified considering that both the human diseases being modeled have febrile seizures and this appears to be a change in neural excitability.

Minor comments:

Figure 4: it is not clear if the scale bar in panel A is mislabeled and should read mV on the y-axis, or if this is a scale bar for the temperature change. There appears to be a stray horizontal line in panel B. Also, the use of an oval and two converging lines to indicate the region that is expanded in panels Ai-Ci is confusing (especially those converging lines). I would recommend including an intermediate time scale for these data that more clearly shows baseline activity, and using just a thick solid line underneath the trace to indicate what time region is going to be expanded rather than the circle and converging lines

Figure 6 (and to a lesser extent Figure 5): The time scale of the voltage protocols and the traces illustrated has been stretched to not match up. It is clear that the capacitance transients for the offset of the voltage steps do not line up, this should be corrected to ensure that the timescales are correct.

Finally, in the discussion the authors describe these mutations as causing a gain-of-function. I understand that some publications in this field use this language very liberally to describe mutations that cause channels to pass more current. This is a very minor pet peeve of mine, but to me a change in deactivation is not a gain of function for the channel, especially since both mutations appear to cause the channels to activate more slowly when heated and cause neurons to spike less. So, the authors are free to use this language if they want, it is not wrong, per se, but I am trying my best to stop the use of gain-of-function for changes like this.

Reviewer 2:

The paper titled “Seizure phenotype and underlying cellular defects in Drosophila knock-in models of DS (R1648C) and GEFS+ (R1648H) SCN1A epilepsy” is focused on characterizing two mutations at the same position in SCN1A, R1648C and R1648H, and their role in epilepsy. R1648C is associated with the severe disorder Dravet syndrome while R1648H is associated with a milder disorder known as GEFS+. The approach being taken to uncover a molecular understanding of the genetic basis for epilepsy is a major strength of this work because much remain unknown about the familial factors that define that disorder. There are several other intriguing aspects of this work including an innovative the use of CRISPR knock-ins. There are also surprising results that both moderate and severe amino acid changes produced similar phenotypes in lifespan and seizure measurements. Moreover, the authors speculate that this similar effect could be a result of the genetic background in which the mutations occur in patients and not the molecular changes themselves, a plausible scenario given the complex nature of epilepsy. There are however also some concerns raised by the paper that if addressed, would further enhance the impact of the work. These include the need to present either genetic or pharmacological rescue data to establish that the effect seen is solely the result of the knock-in and not a secondary effect from elsewhere. Overall, there is strong enthusiasm for this work.

Major Comments:

- The control experiments done on the CRISPR lines to show that the amino acid residue and not the CRISPR construct are responsible for the effects seen alleviate most concerns regarding the specificity of the lines. In order to completely rule out non-specificity or the presence of a secondary effect, there is a need to do a genetic or pharmacological rescue experiment restore the WT state in the R-C and R-H knock-in. The authors should either present these data or clearly address the need for such an experiment.

- Electrophysiological recordings in GABAergic neurons revealed sustained depolarization events and altered firing frequency in the mutants. As written, it indicates, judging by sustained depolarization (Fig. 3D), that R-H appears to exhibit the stronger effect relative to R-C (somewhat at 30oC but more clearly at a shift back to 22oC) even though R-C is the more severe variant. The authors should address this point.

- Also from Fig 3A-D above, there appears to be a penetrance effect at 22oC in the sustained depolarization phenotype shown by R-H and R-C such that a minority of neurons exhibit that effect. The authors should better articulate why the mutations produce that variability.

Minor Comments:

- There needs to be additional information providing a clear rationale for choosing high temperature (40oC) as the second incubation temperature. The writing appears to presume an understanding of the assay and the role of higher temperature in increasing seizure probability by the reader, which is fair, but the temperature paradigm stills need to be more fully described.

- Also following up on the temperature changes, in Fig. 2 the two temperatures used were 22oC and 40oC (although the effective temperature was stated to be around 33oC). However, in the synaptic physiology experiments, the temperatures used were 22oC and 30oC. The authors should correlate between these two sets of temperatures and address for instance whether the temperature used in Fig. 3 is still aimed at the same goal of analyzing seizure-like events but at the synaptic physiology level.

- On Pg. 10 under the Results section on seizure-like behavior. Add to the first sentence a brief statement describing why the seizure-like behavior is being used in that context. This is important since it is the first time it would be introduced in the Results. And while seizures are described in the Introduction, those references refer to a more clinical setting. If a correlation is being drawn to the clinical setting, then that needs to be stated clearly.

- On Pg. 13, the authors note that “Quantitative analysis of these events demonstrate that at 22˚C, sustained depolarizations and post-stimulus depolarizations occurred in a minority of mutant neurons but were never seen in control neurons”. However, looking at Fig. 3D, at least upon a return to 22oC, the sustained depolarization is not in a minority of R-H neurons but a majority. Please revisit and revise.

- Page 4 (last paragraph of the Intro), there is an errant question mark, please remove.

- Make the font size of the figure legends larger.

## Author Response

Dear Dr. Santhakumar,

We thank you and the reviewers for the thoughtful comments and helpful suggestions on the paper. We apologize for the delay in submitting the revisions.

The summary comments as well as the specific comments posed by both reviewers are addressed below. We have revised the paper, the figures, and have added in new information and discussion items as requested.

We believe these changes have strengthened the manuscript and hope that it is ready for publication.

Sincerely,

The Authors

Summary Comments

The study uses CRISPER-Cas9 to develop Drosophila mutants of SCN1A relevant to Dravet and GEFS+ epilepsy syndromes and characterizes the model. The reviewers are in agreement that the experiments are well-designed and rigorously conducted and provides new information regarding the basic physiology and behaviors which are of interest to the field. While the lack of mechanistic examination was noted as a concern, the thorough characterization of the novel model presented here was found to have considerable merit. There were, however, some specific concerns noted by the reviewers which need to be adequately addressed.

1. A more extensive reporting of the intrinsic active and passive properties of neurons under control and hyperthermic conditions is needed to enable correlating changes in sodium channel deactivation with neuronal firing. The potential for compensatory changes to maintain cell intrinsic physiology should be considered and presentation of the full complement of intrinsic physiology data would help identify potential compensatory changes.

To more fully describe the passive and active membrane properties at both low and high temperatures we have added Table 1 (page 16-17) which displays values for input resistance and cell capacitance. We measured the threshold, amplitude, half-width, and maximum rise slope for the first evoked action potential in each cell, the spike frequency adaption ratio (at half-maximal firing, first interspike interval over final interspike interval), and peak instantaneous firing frequency at baseline 22˚C, during heating to 30˚C, and after cooling back to 22˚C for each genotype. There were no significant differences between genotypes for any of these characteristics. While these properties were important to look at, the data continues to support an interaction of sodium channel properties with heat-induced changes to other aspects of cellular physiology that result in aberrant neuronal properties in mutant cells. For example, increased conductance of calcium channels at elevated temperatures could decrease the ability of potassium channels to repolarize mutant neurons following increased conductance of sodium channels due to the mutation.

2. While genetic or pharmacological rescue experiments demonstrating restoration of the WT state in knock-in mice would be ideal to ensure specificity, the authors should, minimally, discuss the need for such an experiment as a potential weakness.

While rescue experiments would indeed be interesting, the method used to generate the knock-in flies in the study already provides strong evidence that the alterations in behavior and firing properties of inhibitory neurons are the result of the mutation knocked into the sodium channel. In the present study, a control (R-R) line was made with the same CRISPR strategy, gRNA, and repair template as two R-C and R-H mutant lines. The only difference was the modification of the codon at the 1648th amino acid position. In addition, the two-step CRISPR strategy used an intermediate line that was identical for the control and mutant lines, in which exon 30 of Para was excised and replaced by DSRed flanked by C. elegans sequences. These worm sequences used to target excision in the second step are very specific since they are not present elsewhere in the genome and sequencing through the excision sites in all lines revealed identical sequences. The parallel generation of the control and two mutant lines, and comparison between them showing similar alterations in both mutant lines compared to control, strongly supports the conclusion that the differences observed are due to the specific mutations and not to other nonspecific CRISPR related alterations. More detail has been added to the first paragraph on page 10 clarifying the presence and role of the C. elegans sequences and in the discussion on page 23. In addition, we have added a sentence in the discussion (page 27) to indicate that further confirmation of the specificity of the mutants could be done by determining if a genetic rescue, using CRISPR to replace the mutation in the R-H and R-C lines, with the control sequence restores wildtype function.

3. In Fig. 3, differences in the firing phenotype between R-C and R-H variants and the variability in penetrance should be discussed.

Based on Chi-squared analysis, there is no significant difference between R-C and R-H mutants in the percentage of cells with sustained depolarizations and post-stimulus sustained depolarizations. This has now been stated clearly in the Results section describing Figure 3. The observance of sustained depolarizations in only 1/7 LNs in each of the mutant lines at 22˚C is most likely due to this being a low frequency event in any one neuron at this temperature. Thus, there is a low probability that an event will be detected during the one or two trials during which the data is collected. If this was true then increasing the number of trials in each cell at 22˚C would increase the percentage of LNs in which at least one event was detected. At high temperature, the majority of neurons in both mutant lines exhibit these events demonstrating that the alteration that leads to these existed in all the neurons but was only manifest at high temperature. It is possible that larger sample sizes could even out some of these smaller changes between R-C and R-H mutants. It is also possible that there are different subclasses of LNs with distinct distribution of sodium channels in the cell, such that some are more susceptible to the mutation resulting in sustained depolarizations at 22˚C. A new paragraph has been added to the Discussion (page 24) reflecting on these points.

4. The long duration traces in figure 4 need to be better explained. It is unclear what the lines signify and basal frequency and action potential characteristics appear different between genotypes and need to be quantified.

We have revised Figure 4 significantly to clarify what the long duration traces represent and to show expanded segments to further illustrate the underlying features including membrane oscillations and action potentials. The long traces (4A, 4B, 4C) each represent a continuous 14 minute whole cell recordings from an individual inhibitory neuron of the indicated genotype during which the tissue was heated and cooled. The recordings illustrate the underlying oscillations in membrane potential with burst firing riding on the oscillation peaks (see Fig. 4Ai-Ciii). There was substantial variability in overall firing frequency between cells within each genotype, however, statistical analysis revealed no significant difference in burst frequency, action potential threshold, action potential amplitude, and action potentials per burst between genotypes. This data is now included in Table 2 (page 19-20). There was however, as previously noted, an increase in the frequency of long depolarizations in R-H mutants at elevated temperature (Fig. 4D), associated with a significant decrease in firing frequency in R-H neurons at elevated temperatures (Fig. 4F).

5. The apparent change in latency to response onset observed in Fig. 5 needs to be quantified and discussed.

The issues with inadequate space-clamp in voltage-clamp protocols do lead to a delay between stimulus onset and sodium current activation. However, statistical comparisons revealed no significant temperature- or genotype-induced change in latency to response onset. Therefore, new sample traces have been chosen so as to not be misleading on this point (page 21) and additional wording has been added to the paragraph in page 21 describing the space-clamping delay.

6. The high temperature used (40 deg) should be adequately justified.

The water bath temperature of 40˚C was used for the seizure assay as it resulted in clear seizure behavior in all mutant flies tested within two minutes of vial submersion. Control flies did not display seizure behavior under these conditions. While it was not possible to easily measure internal temperature in individual flies as one can in a mouse, we did measure the temperature inside the vial during heating. This graph (Figure 2Bi), demonstrates a maximum temperature of 34˚C at the end of the 2-minute trial. All flies survived the heating process and the seizure behavior in the mutants ceased when the vials were removed from the heating bath and returned to room temperature. The maximum elevated temperature used for electrophysiological recordings was 30˚C - a vial temperature at which there was a 60% seizure probability in mutant flies and that was compatible with maintaining long lasting single cell recordings. Higher temperatures resulted in degradation of the integrity of the recordings. Additional details regarding the temperature for the behavior assay and electrophysiology were added to the relevant Methods (page 6) and Results (page 10) sections.

7. Several minor issues with the figures, raised by the reviewers, need to be addressed to improve clarity.

The reviewers made very helpful comments on the figures and the changes have been addressed as detailed below.

Reviewer 1:

In this work the authors have engineered novel models of two human epilepsies, Dravet syndrome and GEFS+, which can both be caused by mutations of the same gene but have different outcomes. They have accomplished this using CRISPR-Cas9 to insert point mutations into the Drosophila para gene. They have measured seizures, early mortality, and changes in single neuron excitability and sodium currents using whole cell electrophysiology. These experiments are well-designed and rigorous. While these experiments don’t reveal mechanistic differences between these two epilepsy syndromes, this work is worthy of publication after revision and will be of interest to the readership of eNeuro.

Major comments:

The first point on which this manuscript could be improved is a deeper analysis of data on their most important findings. The report changes in action potential firing under control and hyperthermic conditions (Fig 3 and 4), and changes in sodium channel deactivation that could underlie this (Fig 6). However, a great deal more data should be presented from these experiments (which does not require performing more experiments). Action potential rise, decay, spike peak amplitude, and spike half width, action potential voltage threshold, peak instantaneous frequency, and spike frequency adaptation would all be of interest and could help link functional changes in spike behavior with changes in sodium channel deactivation. Basic intrinsic properties such as input resistance and resting membrane potential, as well as these spike properties, compared between normo- and hyper-thermic conditions could indicate how sodium channel changes alter baseline excitability or how neurons may be compensating for altered activity.

To more fully describe the passive and active membrane properties at both low and high temperatures we have added Table 1 (page 16-17) which displays values for input resistance and cell capacitance. We measured the threshold, amplitude, half-width, and maximum rise slope at room temperature and high temperature for the first evoked action potential fired, the spike frequency adaption ratio (at half-maximal firing, first interspike interval over final interspike interval), and peak instantaneous firing frequency at baseline 22˚C, during heating to 30˚C, and after cooling back to 22˚C for each genotype. There were no significant differences between genotypes for any of these characteristics. While these properties were important to look at, the data continues to support an interaction of sodium channel properties with heat-induced changes to other aspects of cellular physiology that result in aberrant neuronal properties in mutant cells. For example, increased conductance of calcium channels at elevated temperatures could decrease the ability of potassium channels to repolarize mutant neurons following increased conductance of sodium channels due to the mutation.

Neurons appear to hyperpolarize during hyperthermia (Fig 3 and 4). Is this a consistent change?

The hyperpolarization of membrane potential during spontaneous firing at hyperthermia is consistent across cells and genotypes. Interestingly, resting membrane potential initially depolarizes as the chamber temperature increases. As the temperature further increases and firing ceases, the RMP hyperpolarizes, then recovers as the chamber and cell cools. This pattern is consistent with other papers including Sun et. al. 2012. While RMP did not recover perfectly during the heating protocols, data we did gather indicates that R-H mutants had a significant depolarizing shift in RMP at elevated temperature (Table 2). A paragraph has been added to the Results section (page 24) mentioning the change in RMP over temperature changes and the depolarizing shift in R-H, and Table 2 has been updated to clearly display this information.

In Figure 4, the long duration recording traces (panels A-C) presented are difficult to interpret. Is baseline spontaneous AP frequency changed? What are the properties of those action potentials? Do the bursts describe in Table 1 have the same duration and number of spikes across genotypes?

We have revised the figure significantly including adding inlays on an expanded time scale to illustrate the pattern of burst firing at all three temperatures for each of the 3 genotypes. An expanded, and now supplemental Table 2 displays no differences in burst threshold, amplitude, or action potentials per burst. An increase in sustained depolarization frequency in R-H neurons (Fig 4D) resulted in decreased firing frequency in R-H neurons at elevated temperature (Fig 4F).

Both mutant examples appear to exhibit periods of depolarization at 22 degrees. Is this true of most or all mutant neurons?

LNs of both genotypes occasionally exhibited depolarizations longer than 100ms, even at room temperature. This has been stated in the text, and shown in Fig. 4E. 4/9 R-C LNs and 6/9 R-H LNs exhibited sustained depolarizations at 22 degrees. Interestingly, 1/9 R-R LNs also had one single sustained depolarization over 100ms at room temperature, and one at elevated temperature. The incidence of cells exhibiting sustained depolarization was not different between genotypes at 22˚C based on a Chi-squared analysis. Additionally, the sustained depolarizations occurred so infrequently at 22˚C in all genotypes that the overall firing frequency within bursts at room temperature was not significantly different between any of the genotypes. At elevated temperature, the frequency of sustained depolarizations increased in the mutant R-H and R-C LNs. There was no change in control LNs. At high temperature the difference between R-H mutant and control neuron was significant (4D). This further contributes to a decreased firing frequency that is significantly reduced in R-H LNs.

Finally, in Fig 5 the authors mention the clear change in delay to onset of sodium current in mutant neurons during hyperthermia. This seems like an important change that should be quantified considering that both the human diseases being modeled have febrile seizures and this appears to be a change in neural excitability.

Our text led to a misunderstanding regarding this topic. While there were space-clamping issues at all temperatures in all genotypes leading to a delay in activation of the sodium currents, there was no difference in this delayed activation as a function of genotype or temperature. A sentence in the paragraph regarding this figure (page 21) has now included the words “the significant delay between the stimulus onset and sodium current activation at all recording temperatures is due to the recording electrode being located on the LN cell body...” Additionally, new traces have been chosen for Figure 5 that show similar delays from the onset of the stimulus to activation of the inward sodium current (page 21).

Minor comments:

Figure 4: it is not clear if the scale bar in panel A is mislabeled and should read mV on the y-axis, or if this is a scale bar for the temperature change. There appears to be a stray horizontal line in panel B. Also, the use of an oval and two converging lines to indicate the region that is expanded in panels Ai-Ci is confusing (especially those converging lines). I would recommend including an intermediate time scale for these data that more clearly shows baseline activity, and using just a thick solid line underneath the trace to indicate what time region is going to be expanded rather than the circle and converging lines

Thank you for pointing out some of the confusion surrounding this figure. Figure 4 has been redone to include inlays of burst firing at room temperature, during heating, and post-cooling for all three genotypes, and two different scale bars, one for the long traces and one for the expanded traces are shown in A and Ai.

Figure 6 (and to a lesser extent Figure 5): The time scale of the voltage protocols and the traces illustrated has been stretched to not match up. It is clear that the capacitance transients for the offset of the voltage steps do not line up, this should be corrected to ensure that the timescales are correct.

Thank you for pointing this out. This has been corrected (page 21).

Finally, in the discussion the authors describe these mutations as causing a gain-of-function. I understand that some publications in this field use this language very liberally to describe mutations that cause channels to pass more current. This is a very minor pet peeve of mine, but to me a change in deactivation is not a gain of function for the channel, especially since both mutations appear to cause the channels to activate more slowly when heated and cause neurons to spike less. So, the authors are free to use this language if they want, it is not wrong, per se, but I am trying my best to stop the use of gain-of-function for changes like this.

We agree that changing the language to be more specific is beneficial. We have taken out references to “gain of function” in regards to the findings of this paper. These replacements occurred in the Significance Statement (page 2) and Conclusion (page 28 and page 30).

Reviewer 2:

The paper titled “Seizure phenotype and underlying cellular defects in Drosophila knock-in models of DS (R1648C) and GEFS+ (R1648H) SCN1A epilepsy” is focused on characterizing two mutations at the same position in SCN1A, R1648C and R1648H, and their role in epilepsy. R1648C is associated with the severe disorder Dravet syndrome while R1648H is associated with a milder disorder known as GEFS+. The approach being taken to uncover a molecular understanding of the genetic basis for epilepsy is a major strength of this work because much remain unknown about the familial factors that define that disorder. There are several other intriguing aspects of this work including an innovative the use of CRISPR knock-ins. There are also surprising results that both moderate and severe amino acid changes produced similar phenotypes in lifespan and seizure measurements. Moreover, the authors speculate that this similar effect could be a result of the genetic background in which the mutations occur in patients and not the molecular changes themselves, a plausible scenario given the complex nature of epilepsy. There are however also some concerns raised by the paper that if addressed, would further enhance the impact of the work. These include the need to present either genetic or pharmacological rescue data to establish that the effect seen is solely the result of the knock-in and not a secondary effect from elsewhere.

Overall, there is strong enthusiasm for this work.

Major Comments:

- The control experiments done on the CRISPR lines to show that the amino acid residue and not the CRISPR construct are responsible for the effects seen alleviate most concerns regarding the specificity of the lines. In order to completely rule out non-specificity or the presence of a secondary effect, there is a need to do a genetic or pharmacological rescue experiment restore the WT state in the R-C and R-H knock-in. The authors should either present these data or clearly address the need for such an experiment.

While rescue experiments could be done, the method used to generate the knock-in flies in the study already provides strong evidence that the alterations in behavior and firing properties of inhibitory neurons are the result of the mutations knocked into the sodium channel. The control (R-R) line was made with the same CRISPR strategy, gRNA, and repair template as two R-C and R-H mutant lines. The only difference was the modification of the codon at the 1648th amino acid position. In addition, the two-step CRISPR strategy used an intermediate line that was identical for the control and mutant lines, in which exon 30 of Para was excised and replaced by DSRed flanked by C. elegans sequences. These worm sequences used to target excision in the second step are very specific since they are not present elsewhere in the genome and sequencing through the excision sites in all the lines revealed identical sequences. The parallel generation of the control and two mutant lines, and comparison between them showing similar alterations, although there are some differences in magnitude, in both mutant lines compared to control, strongly supports the conclusion that the differences observed are due to the specific mutations and not to other nonspecific CRISPR related alterations. More detail has been added to the first paragraph on page 10 clarifying the presence and role of the C. elegans sequences. In addition, we have added a sentence in the discussion to indicate that further confirmation of the specificity of the mutants could be done by determining if a genetic rescue, such as using CRISPR to place the exon containing the mutation in the R-H and R-C lines with the control exon, restores wildtype function.

- Electrophysiological recordings in GABAergic neurons revealed sustained depolarization events and altered firing frequency in the mutants. As written, it indicates, judging by sustained depolarization (Fig. 3D), that R-H appears to exhibit the stronger effect relative to R-C (somewhat at 30oC but more clearly at a shift back to 22oC) even though R-C is the more severe variant. The authors should address this point.

Based on Chi-squared analysis, there is no significant difference between R-C and R-H mutants in the % of cells with sustained depolarizations and post-stimulus sustained depolarizations. This has been stated clearly in the Results section describing Figure 3. More generally, an entirely new paragraph has been added to the Discussion (page 24) describing the differences seen in only one, but not both, mutant lines. While we point out the similarities in R-C and R-H in the fly model particularly in behavior and the direction of alterations in electrophysiological properties including sustained depolarization that increase in frequency at elevated temperature, we also note that R-H does result in more pronounced changes in several properties than R-C including the firing frequency and the more depolarized resting potential and more hyperpolarized activation threshold. Surprisingly, these changes did not result in a more severe disease phenotype based on the measures we analyzed. It seems that the changes from controls seen in both mutant lines, namely hyperpolarized deactivation threshold and increased incidence of sustained depolarizations, had the greatest impact on behavior.

- Also from Fig 3A-D above, there appears to be a penetrance effect at 22oC in the sustained depolarization phenotype shown by R-H and R-C such that a minority of neurons exhibit that effect. The authors should better articulate why the mutations produce that variability.

The observance of sustained depolarizations in only 1/7 LNs in each of the mutant lines at 22˚C is most likely due to this being a low frequency event in any one neuron at this temperature. Thus there is a low probability that an event will be detected during the one or two trials during which the data is collected. If this was true then increasing the number of trials in each cell at 22˚C would increase the percentage of LNs in which at least one event was detected. At high temperature the majority of neurons in both mutant lines exhibit these events demonstrating that the alteration that leads to these existed in all the neurons but was only manifest at high temperature. It is possible that larger sample sizes could even out some of these smaller changes between R-C and R-H mutants. It is also possible that there are different subclasses of LNs with distinct distribution of sodium channels in the cell, such that some are more susceptible to the mutation resulting in sustained depolarizations at 22˚C. A new paragraph has been added to the Discussion (page 24) reflecting on these points.

Minor Comments:

- There needs to be additional information providing a clear rationale for choosing high temperature (40oC) as the second incubation temperature. The writing appears to presume an understanding of the assay and the role of higher temperature in increasing seizure probability by the reader, which is fair, but the temperature paradigm stills need to be more fully described.

The water bath temperature of 40˚C used for the seizure assay as it resulted in clear seizure behavior in all mutant flies tested within two minutes of vial submersion. Control flies did not display seizure behavior under these conditions. While it was not possible to easily measure internal temperature in individual flies as one can in a mouse, we did measure the temperature inside the vial during heating. This graph (Figure 2Bi), demonstrates a maximum temperature of 34˚C at the end of the 2-minute trial. All flies survived the heating process and the seizure behavior in the mutants ceased when the vials were removed from the heating bath and returned to room temperature. The elevated temperature used for evoked electrophysiological recordings was 30˚C - a vial temperature at which there was∼ 50-60% seizure probability in mutant flies and that was compatible with maintaining long lasting single cell recordings. During recording of spontaneous activity, cells ceased firing before 30˚C, so the minute of heating between 26˚C and 29˚C was used for data analysis. Higher temperatures during the whole cell recordings resulted in more rapid degradation of the tissue making it difficult to maintain a stable recording for the time required to evaluate the effect of ramping up and down the temperature. Additional details regarding the temperature for the behavior assay and electrophysiology were added to the relevant Methods and Results sections.

- Also following up on the temperature changes, in Fig. 2 the two temperatures used were 22oC and 40oC (although the effective temperature was stated to be around 33oC). However, in the synaptic physiology experiments, the temperatures used were 22oC and 30oC. The authors should correlate between these two sets of temperatures and address for instance whether the temperature used in Fig. 3 is still aimed at the same goal of analyzing seizure-like events but at the synaptic physiology level.

This is an important point to clarify. while the seizure behavior assay used a water bath at 40˚C, a maximum recording chamber temperature of 30˚C was used for elevated temperature electrophysiological recordings. 30˚C correlates to an in-vial temperature at which many mutant flies seized in the seizure behavior assay, and also was the highest chamber temperature at which quality recordings could be made. For one set of electrophysiology experiments - the spontaneous firing traces - neurons stopped firing before 30˚C, so the 60 seconds of heating corresponding to the chamber at 26˚C - 29˚C was used. Additional comments were added to the text throughout the Methods and Results sections.

- On Pg. 10 under the Results section on seizure-like behavior. Add to the first sentence a brief statement describing why the seizure-like behavior is being used in that context. This is important since it is the first time it would be introduced in the Results. And while seizures are described in the Introduction, those references refer to a more clinical setting. If a correlation is being drawn to the clinical setting, then that needs to be stated clearly.

Thank you for this suggestion. We added a statement clarifying that the water bath is to simulate febrile conditions that induce seizures in individuals with GEFS+ and DS.

- On Pg. 13, the authors note that “Quantitative analysis of these events demonstrate that at 22˚C, sustained depolarizations and post-stimulus depolarizations occurred in a minority of mutant neurons but were never seen in control neurons”. However, looking at Fig. 3D, at least upon a return to 22oC, the sustained depolarization is not in a minority of R-H neurons but a majority. Please revisit and revise.

We have now clarified on page 14 that the “minority” of mutant neurons displayed sustained depolarizations at the 22˚C pre-heating, that the majority have these at high temperature and there is some but not complete recovery with the incidence of sustained depolarizations decreasing but not returning fully to pre-heating levels during the 5 minute cooling period.

- Page 4 (last paragraph of the Intro), there is an errant question mark, please remove.

The typo was removed.

- Make the font size of the figure legends larger.

We have increased the font size from 8 to 10
